# Therapeutic Alliance as Active Inference: The Role of Therapeutic Touch and Synchrony

**DOI:** 10.3389/fpsyg.2022.783694

**Published:** 2022-02-17

**Authors:** Zoe McParlin, Francesco Cerritelli, Karl J. Friston, Jorge E. Esteves

**Affiliations:** ^1^Foundation COME Collaboration, Clinical-Based Human Research Department, Pescara, Italy; ^2^Wellcome Centre for Human Neuroimaging, Institute of Neurology, London, United Kingdom; ^3^Malta ICOM Educational Ltd., Gzira, Malta; ^4^Research Department, University College of Osteopathy, London, United Kingdom

**Keywords:** therapeutic alliance, active inference, affective touch, synchrony, predictive processing, allostasis, free-energy principle, perinatal care

## Abstract

Recognizing and aligning individuals’ unique adaptive beliefs or “priors” through cooperative communication is critical to establishing a therapeutic relationship and alliance. Using active inference, we present an empirical integrative account of the biobehavioral mechanisms that underwrite therapeutic relationships. A significant mode of establishing cooperative alliances—and potential synchrony relationships—is through ostensive cues generated by repetitive coupling during dynamic touch. Established models speak to the unique role of affectionate touch in developing communication, interpersonal interactions, and a wide variety of therapeutic benefits for patients of all ages; both neurophysiologically and behaviorally. The purpose of this article is to argue for the importance of therapeutic touch in establishing a therapeutic alliance and, ultimately, synchrony between practitioner and patient. We briefly overview the importance and role of therapeutic alliance in prosocial and clinical interactions. We then discuss how cooperative communication and mental state alignment—in intentional communication—are accomplished using active inference. We argue that alignment through active inference facilitates synchrony and communication. The ensuing account is extended to include the role of (C-) tactile afferents in realizing the beneficial effect of therapeutic synchrony. We conclude by proposing a method for synchronizing the effects of touch using the concept of active inference.

## Introduction

The therapeutic alliance (TA) is a collaborative working relationship between a clinician and patient iand is a critical component of person-centerd care because it contributes to positive clinical outcomes across multiple healthcare disciplines (Miciak et al., 2019; Ryu et al., 2021). Recognizing, gaining trust and aligning an individual’s unique beliefs or “priors” through cooperative communication is crucial in patient management and establishing a beneficial TA (Kinney et al., 2018). A successful TA has been shown to improve overall patient satisfaction, quality of life as well as both increase both physical and mental treatment outcomes (Ferreira et al., 2013; Fuentes et al., 2014; Manzoni et al., 2018). The TA alliance is often modeled from the working alliance described by Bordin (1979), which includes the collaborative agreement on goals, tasks, and the development of successful relationships and cooperative communication. In order to achieve cooperative communication individuals will often attempt to selectively gather evidence to align and synchronize their mental states with another individual’s narrative (Vasil et al., 2020). A successful TA, mutual agreement and therefore alignment on clinical goals has been suggested as to why patients report an increased self efficacy, participation and adherence to exercises prescribed (Babatunde et al., 2017; Kinney et al., 2018).

Active inference (AI) is an empirical integrative model that has been proposed to explain the dynamics and biobehavioral mechanisms of cooperative communication (Vasil et al., 2020). An effective method for establishing this cooperative alliance—and potential synchrony relationship—is through ostensive cues generated by repetitive coupling during physical touch (Csibra, 2010). Affectionate (affiliative) touch is unique in its ability to foster communication, interaction, and various therapeutic benefits, for example in the context of perinatal care, it contributes to an infant’s development, both neurophysiologically and behaviorally (Carozza and Leong, 2021).

Synchrony is defined as the interpersonal coordination of behavioral and neurophysiological rhythms that can aid in sensory processing, learning, emotion and arousal regulation, self-regulation, and the establishment of a communicative relationship (Harrist and Waugh, 2002; Beebe et al., 2018; Wass et al., 2020; Carozza and Leong, 2021). Therefore considering the TA principles of agreement on goals, tasks and developing strong relationships a degree of biobehavioral synchrony will often occur within a clinical setting. Additionally, it is human nature to want to share psychological and emotional states with others; e.g., through the structured patterns of communication between mother and infant (Reddy, 2008). These interactions synchronize and individual’s behavior, hidden states (of mind), and biological rhythms, resulting in an intertwined unit akin to a sophisticated waltz (Feldman, 2007; Tschacher and Haken, 2007; Koole and Tschacher, 2016; Kupper et al., 2016; Tschacher et al., 2017). Furthermore, biobehavioral synchrony can be achieved in order to fulfill the role of caregiving when responding to a stimulus for help, i.e., an infant, by activating numerous subcortical regions including the hypothalamus, amygdala, and the dopaminergic reward circuit (Uvnäs-Moberg et al., 2015; Feldman, 2016; Hardin et al., 2020; Carozza and Leong, 2021). The emotional distress exhibited by the infant’s stimulus consequently triggers an empathic response from the caregiver by relating to the infant’s distress, and this can putatively contribute to activations in the anterior insular cortex and mentalizing network (Rizzolatti and Craighero, 2004; Fan et al., 2011). Arguably, motivationally rewarding dyadic exchanges between the infant and their caregivers may result in concurrent activations in the ventral segmental area, nucleus accumbens, insular cortex, amygdala, and hypothalamus and increases in neurochemicals such oxytocin (Li and Fleming, 2003; Ebner et al., 2005; Atzil et al., 2011; Feldman, 2015). A common mode to respond to the stimulus of the infant is often through touch, further increasing the biobehavioral synchrony and therapeutic effectiveness (Kartner et al., 2010; Finset and Ørnes, 2017). Crucially, the limbic system implicated in reward processing regulates key survival and motivation functions, promoting a social life (Sokolowski and Corbin, 2012).

Active inference provides an insight into why human beings choose to synchronize and create working alliances with others in order to minimize the free energy within a bayesian structure through coupling between sentient systems (Friston et al., 2015). AI is a formal account of the precise interactions between one’s internal structure, function, and the external environment. Synchronization occurs because each individual seeks confirmation of their self-fulfilling, genetic, cultural, or social conception of the world, to ensure it is the most probable outcome with the fewest biophysical surprises, increasing efficiency of cooperative communication and subsequently informational and energetic cost (Pezzulo et al., 2018; Ramstead et al., 2018; Vasil et al., 2020). One way to accomplish this effectively is to intentionally synchronize with others through the collection or generation of sensory evidence to reliably confirm and align beliefs, based on the premise that everyone is identical, reducing the potential for surprise and minimizing free energy (Bruineberg et al., 2018; Ramstead et al., 2018; Badcock et al., 2019). In other words, the simplest way to minimize surprise is to selectively engage with people who respond in the same, predictable fashion. As a result, forecasting the most likely narratives that underwrite interpersonal exchange eludes surprises and minimizes prediction errors or mismatches between predicted and actual sensations. This imperative can be seen at many levels: from homeostasis (Koban et al., 2019) to action observation (Kilner et al., 2007; Edwards et al., 2012; Seth and Friston, 2016). Additionally, the individual will be more attuned to the environment, allowing for the learning and consolidation of prior beliefs and subsequent repetition of surprise-minimizing behavior (Friston and Frith, 2015a; Isomura et al., 2019). Arguably, alignment through active inference facilitates synchrony and communication and contributes to changes in a patient’s generative model.

Technically, active inference refers to the inversion of Bayesian (i.e., generative) models of the sensed world, where these models include the consequences of action. Model inversion can then be read as perceptual inference; namely, inferring the (unobservable) causes of (observable) sensations. Because these causes include action, active inference entails policy selection and planning as inference (Attias, 2003; Botvinick and Toussaint, 2012; Mirza et al., 2016; Friston et al., 2017). Mathematically, perceptual inference corresponds to a minimization of variational free energy, while actions are chosen that minimize the expected free energy following an action. In this setting, free energy can be read as scoring the implausibility of (sensory) outcomes, usually described in terms of surprise or prediction errors (Clark, 2013; Hohwy, 2013). Note, that surprise is not a propositional surprise, it is just a mathematical way of describing how unlikely outcome is, given a model that generates or predicts the most likely outcome.

We will use “surprise” in this technical sense in what follows. The expected free energy corresponds to the surprise expected following an action, which can be read as uncertainty. In short, surprise scores how unpredicted a particular outcome is, while uncertainty scores how unpredictable some outcomes will be in the future. Formally speaking, expected free energy is usually decomposed into risk and ambiguity, where risk scores the departure from prior preferences or outcomes that can be regarded as rewarding. This means selecting actions that minimize expected free energy have both pragmatic (goal or reward-seeking) and epistemic (information-seeking) aspects. We will consider both these kinds of affordances in dyadic interactions, with a special focus on touch and interoception.

Physical touch is a common and fundamental method for establishing salience and social relationships, resulting in biobehavioral synchrony (Craig, 2002; Aureli and Presaghi, 2010; Fotopoulou and Tsakiris, 2017). Touch is regarded as a sensory tactile stimulation of multiple myelinated and unmyelinated mechanoreceptors. Low threshold, heavily myelinated AB afferent mechanoreceptors respond to rapid sensory stimulation and discriminate between the physical sensations of touch (Manzotti et al., 2019). In contrast, unmyelinated C-tactile (CT) afferent fibers detect slow, stable, gentle touch and are sensitive to skin temperature, sending signals to the limbic system. Affective touch via tactile stimulation can be viewed as an example of a particular embodied social behavior that maintains homeostasis and influences the perception of—or inference about—the mental states of self and other (Ciaunica et al., 2021). Moreover, it has been speculated that touch may cause a rapid reduction in the activity in the pre-frontal cortex before instantiating a sustained period of pre-emptive homeostatic regulation (Shamay-Tsoory and Eisenberger, 2021). It has also been suggested that the use of social touch decreases the level of effort needed to overcome stress (Coan and Maresh, 2014). When considering the hypothesis that social touch is a distinct mode of touch, it has been suggested that the CT fibers may selectively sample slow sensory fluctuations that are socially salient (i.e., that are generated by the affiliative touch of another person). And determine whether the stimulus is perceived pleasant as it is processed in the interoceptive hierarchy, including the insula (Björnsdotter et al., 2010).

In this paper, we propose a framework for the role of touch in relation to building synchrony to underwrite a therapeutic alliance—in a range of clinical situations—through the lens of AI. We start with an overview of the essential characteristics of how we establish cooperative communication to achieve biobehavioral synchrony via touch. We then discuss how, once established, synchrony is continually developed and how the brain works to overcome the uncertainty (i.e., expected surprise) inherent in the lived world. Subsequently, we will discuss the neurophysiological aspects of synchrony, and apply the concepts to the formation of both dyadic and triadic relations (Trapp et al., 2014). We discuss overcoming uncertainty in the clinical situation before delving into the role of empathy and the use of repetition within the appointment structure. We conclude by discussing the adaptations and synchronization of ideas, emphasizing diagnosis and the process by which patients and practitioners can develop a shared treatment plan.

### The Importance of Positive Connection and How to Foster it

Throughout human evolution, the brain has evolved to acquire and maintain sufficient resources to enable the body to perform the essential (existential) functions of growth, healing, and reproduction required for survival, achieving allostasis in the process (Sterling and Eyer, 1988; Ramsay and Woods, 2014; Barrett et al., 2016; Seth and Friston, 2016). Most of our interactions are motivated by our mutual expectation of cooperative communication and synchrony with a common mode being social touch (Suvilehto et al., 2015). Allostasis is a process by which the brain predictively regulates the body metabolic and energy needs using an internal model of that body in the (Barrett, 2017). It has been proposed that the computational architecture of the brain involved in allostatic active inference processes depend on the default mode network, salience network and the frontoparietal network (Kober et al., 2008; Binder and Desai, 2011; Van den Heuvel and Sporns, 2013; Barrett, 2017). This is demonstrated in infants through cultural learning when they attempt to infer future events or uncertainties based on what trusted others have learned or believed (Harris and Corriveau, 2011; Harris et al., 2017). Cooperative communication helps us become more attuned to our environment, creating priors to predict future events and build a stronger therapeutic alliance. Additionally, achieving allostasis requires a delicate balance of various physiological mediators, including autonomic, inflammatory, metabolic, cortisol, and neuromodulators, to induce transient adaptations (McEwen and Wingfield, 2003; D’Alessandro et al., 2016). Moreover, the brain will prioritize and motivate stimuli in a hierarchical manner in order to explain, infer and later attempt to control their self fulfilling predictions as a result improving communication and biobehavioral synchrony (Sterling, 2014).

This process of allostasis regulation was demonstrated during collaborative human foraging in a stag hunt situation; it was evident that larger groups working cooperatively could achieve greater hunting rewards such as catching a stag than an individual hare could (Tomasello et al., 2012). To secure the stag, they employ “joint attention,” in which two or more individuals work cooperatively on the same problem to achieve a positive (unsurprising) outcome. As a result, they increase their communication efforts and align their mental states, to attempt to offset the risk of trusting others (Tomasello, 1995; Skyrms, 2001). This alignment is evident in increased interpersonal brain synchronization when interacting with individuals you have a higher social bond to, including a successful working alliance, reflecting in the synchrony within the inferior Parietal lobe (Kinreich et al., 2017; Zhang et al., 2018). By hunting the stag, the group achieves increased allostatic regulation—through social and cultural synchrony—as well as the nutritional benefits necessary for survival, motivating them to repeat the collaborative communication method in the future (Yoshida et al., 2008; Devaine et al., 2014; Duguid et al., 2014; Feldman, 2015; Begus et al., 2016). The limbic system, which connects external cues involved in social and emotional processing to cultural behaviors essential for ensuring survival and motivation may also influence the precision of new sensory stimuli when establishing allostatic regulation (Sokolowski and Corbin, 2012; Barrett, 2017). It is critical to infer the “hidden” thoughts of others to engage in cooperative communication and thus synchrony. These inferences (c.f., theory of mind) enable individuals to work together to resolve uncertainty and achieve familiar outcomes. Touch can be viewed as a form of tactile communication and an ostensive cue indicating their partner’s perceptions, thoughts, and feelings about the current context and whether they have shifted with subtle changes the quality of touch accurately inferring different emotions (Hertenstein et al., 2006a,b). Thus, to synchronize and overcome this uncertainty, we frequently rely on and employ specific perceptual and ostensive cues such as gaze, touch, and facial expressions to infer a partner’s intentions and dispositions and, hopefully, better align with them. This connection is frequently strengthened through touch, as seen when romantic couples hold hands, thereby increasing the phenomenon of neural synchrony (Goldstein et al., 2018). The limbic system’s association with touch explains why touch is crucial in developing relationships, alliances and synchrony between individuals. Additionally, we must cultivate self-other discrimination, attribution of agency and interpersonal inference through face-to-face interactions, gaze, ostensive cues, and hand/gesture holding.

### The Importance of Physical Touch in the Development of Interpersonal Connections

Touch can be viewed as key in various situations for the interpersonal inference necessary for establishing interpersonal connections and, potentially, synchrony (Hertenstein et al., 2006a; Fonagy and Allison, 2014). Touch can increase trust, compliance, and generosity; while simultaneously reducing anxiety and threatening negative emotions (Morrison et al., 2010). Additionally, it can be used and manipulated to assist in accurately applying context to infer different hidden thoughts, communicative intentions and at least 6 different emotions of others including love, fear and anger, with between 48 and 83% accuracy (Hertenstein et al., 2006b). The more precise one becomes at inferring others’ thoughts and actions, the more adept one establishes common ground, trust, synchrony, and a beneficial therapeutic alliance. Additionally, interpersonal synchrony between parent and infant is well documented through non-verbal behaviors such as touch, gaze, and voice, particularly in mothers (Feldman, 2016; Fotopoulou and Tsakiris, 2017). Reciprocal somatosensory responses within the orbitofrontal cortex and temporal lobe, but also involving the cingulate cortex, caudate nucleus, preoptic region, lateral hypothalamus, and ventral tegmental area (Rolls, 1999; Kringelbach and Rolls, 2004), as well as electrodermal activity coupling between the ventromedial prefrontal and medial orbitofrontal cortex, may putatively provide the neural correlates for social value within co-operative communications (Kringelbach and Rolls, 2004; Behrens et al., 2008; Ruff and Fehr, 2014; Murray et al., 2015). This is observed in individuals that have the intention to care for others, while also having an established intimate bond, not just between mother and infant. However, with more intimate or significant relationships, the inter-brain synchrony and brain to brain coupling induced by interpersonal touch is enhanced with different activation patterns in the somatosensory, Anterior Cingulate Cortex (ACC) and occipital frontal cortices (McCabe et al., 2008; Gazzola et al., 2012; Goldstein et al., 2018). This is corroborated by observed coupling in partners’ right temporoparietal regions during touch, implying a link between touch, attention orientation, self-other discrimination, and perception (Goldstein, 2017). Additionally, romantic couples exhibit the most significant degree of heart rate and respiration synchrony when one of the partners is in pain. This is possibly due to the other individual needing additional emotional support, most notably through empathy.

Additionally, the sight of a familiar face stimulates an immediate release of oxytocin; however, when the interaction is followed by physical touch, a second wave of prolonged oxytocin is released, cortisol is decreased, and closeness is increased (Uvnäs Moberg et al., 2020). The more “in sync” the interaction and the stronger the bond, the more oxytocin is released (Feldman, 2006). The reaction to a familiar face is due to increased salience due to “affective meaning” based on prior experiences, which causes the individual to recreate the experience, even if they are not fully experiencing it (Roy et al., 2012). As a result, it triggers the release of oxytocin with less significant sensory stimuli such as sight or smell, rather than requiring a more significant sensory stimulus such as physical touch (Uvnäs Moberg et al., 2020). As a result, touch can also enhance bonding, oxytocin release, heart rate, frontal alpha EEG synchrony, and activation of the temporo-parietal regions all building synchrony and a therapeutic alliance. See Quattrocki and Friston (2014) for a discussion of oxytocin in the setting of active inference, attachment and synchronization.

This rapid physiological response caused by synchrony is also seen in the innate, sub-second reaction to proximal touch, which mothers give to their six-week-old infants in the form of body contact, touch, or physical stimulation. Proximal touch was found to be the preferred mode of communication, eclipsing audio and visual communication (Kartner et al., 2010). Additionally, 65 percent of face-to-face play involves physical contact, which helps alleviate stress through social buffering (Stack and Muir, 1990; Kikusui et al., 2006). After one year, an infant who is quickly picked up when crying has been shown to learn to self-soothe and thus cry less (Benoit, 2004). The intuitive, less than one second contingent responsiveness from a mother to attend to her infant, is thought to originate from the hypothalamic-pituitary-adrenal axis, which heightens their awareness of their infant (Kartner et al., 2010; Barrett and Fleming, 2011), confirming once again that touch is an instinctive ostensive cue in the development of communication and synchrony.

### *In utero* Initiation of Synchrony

The earliest manifestations of synchrony occur during the brain and nervous system development of a fetus *in utero*. Throughout pregnancy, a direct active bidirectional flow of environmental and interoceptive sensory signals is transmitted to the fetus via the mother’s heart rate, movement, touch, stress, and social cues, which is passed on to the fetus as the mother attempts to maintain allostasis for their mutual health (Ciaunica et al., 2021). A disruption in the allostasis of the stress mediators or their excessive use can result in sensitization overload, resulting in physiological symptoms such as chronic pain (McEwen et al., 2015). Bilateral cardiac synchrony is evident as early as prenatal development; when maternal stress, vagal tone, and heart rate variability inter-beat rate increase, an increased mean fetal heart rate within one second of the mothers, also increases (Feldman et al., 2011). When the mean maternal heart rate decreases at night, the fetal heart rate decreases to reflect a bilateral somatosensory response and coupling of the electrodermal activity (Ivanov et al., 2009; Feldman et al., 2011). In active (interoceptive) inference, heart rate is a key realization autonomic reflexes that rest upon inferring the right kind of sympathetic—or parasympathetic—context (Seth and Friston, 2016). The interaction between mother and infant allows the infant to align the biological rhythm with that of others. Infants, it is hypothesized, will initially respond to the “similar to me” theory to learn what it is like to be a member of the species, influenced by their bond with their mother: thereby creating a blueprint template for future social experiences, correlating with a hierarchical predictive processing model (Atzil et al., 2014; Shimon-Raz et al., 2021).

Throughout pregnancy, the mother exerts control over her interactions with the external world, suggesting it lays the groundwork for the infant’s homeostatic mechanism and future priors particularly in regard to food preferences and flavors (Mennella et al., 2001). Continuous bilateral interaction primes the infant’s homeostatic regulation, acclimates it to direct social proximity, and equips it with the tools necessary to adapt and respond to the temporal dimension of social stimulus before they are born (Akers et al., 2008; Feldman, 2017). This experience of co-regulation lays the groundwork for the infant’s ability to engage in social interactions such as intimacy, empathy, and inferring hidden thoughts. By governing and establishing the initial priors for numerous internal models, the fetus conserves energy while developing by avoiding the active maintenance of homeostasis, while ensuring allostasis. However, it has been demonstrated that a fetus can exert influence over allostatic control and kick the uterine wall when in pain or dissatisfaction, eliciting a response from the mother. It teaches the fetus through repetition that the mother will react in a certain way to the kick to control and satisfy its physiological predictions and preferences within the dyad (Ciaunica et al., 2021).

### Post-natal Development of Synchrony Through Touch

Infants continue to rely on their caregivers for survival following birth, which correlates with the infant constructing their conceptual model of the world around them based on the temporal dimensions of the social dyads established by their caregiver through contingency detection (Tarabulsy et al., 1996). Infants instinctively maintain synchrony and thus regulation with the mother after birth, as they participate in co-homeostatic and allostatic regulation during co-embodiment (Ciaunica et al., 2021). Infants who are highly attuned to their mothers frequently allow them to learn in detail from their early interactions and their mothers’ unique biological rhythms and priors how to socialize, empathize, and infer the emotions of others (Feldman, 2007). This bidirectional interaction results in forming a “homeostatic fetus and an allostatic mother” (Ciaunica et al., 2021). Synchrony between mother and new-born infant is frequently established with the mother’s sensitive behavior to maximize the infant’s ability to detect differences in their environment, other people’s behavior, and their own behavior (Tarabulsy et al., 1996). Additionally, it is possible to argue that the infant developed and survived birth trauma due to their mother’s care, further enhancing the salience and weighting of her priors relative to other stimuli.

Often, the first communication and connection with one’s mother occurs immediately after birth through skin-to-skin contact. This skin-to-skin contact is recommended because touch has been shown to help reset neural oscillations and align them with their parents’ oscillation patterns in their neocortex particularly the pregenual ACC, all while reducing overall stress (Takahashi et al., 2011; Wass et al., 2020). Additionally, comforting touch is believed to provide more physical and mental relief from stress than self-care and would contribute to recovering from the distress of birth (Shamay-Tsoory and Eisenberger, 2021). When combined with other forms of ostensive cues, afferent stimulation from the skin-to-skin contact may contribute to biobehavioral synchrony (Carozza and Leong, 2021). Additionally, the mother’s touch and physical body can be viewed as a proactive external regulatory mechanism that shapes the infant’s neurobiological, sensory, emotional, physical, and social systems in response to contextual cues (Hrdy, 1999).

Increased lateralization, particularly in the left frontal hemisphere, results in increased maternal warmth, physiological regulation, and the development of specific coordinated social behaviors and bonding (Feldman et al., 2006; Hardin et al., 2020). The co-homeostasis established *in utero* is maintained postnatally through touch, including breastfeeding and skin to skin contact, which increases sensory oxytocin release in both mother and infant. Additionally, a caregiver’s touch frequently induces a state of calm alertness, which frequently signals to the infant that the caregiver is attempting to communicate (Harrison et al., 2000; Carozza and Leong, 2021). When new-born infants coordinate their limb movements to the rhythm of the adult’s speech, this intention to communicate is reciprocated (Condon and Sander, 1974). A strong bond between parent and child will amplify the parasympathetic response to affectionate touch (Aguirre et al., 2019).

### Touch and Exploration aid in Establishing a Reference for Homeostasis

We frequently rely on touch to investigate, confirm, and discriminate stimuli in complex or unfamiliar environments rather than initiate communication. This is done to amass more precise sensory evidence and establish reference values and priors for various homeostatic mechanisms to enable regulation in the future. These baseline references are frequently established during childhood. Creating a prior of this sort enables users to prioritize specific data to improve prediction, increase synchrony, and reaffirm their priors through homeostasis. This can be observed as early as 8–10 weeks gestation, when a fetus begins to use their fingers to touch their lips and face, coinciding with the development of sensory nerves to that area, to explore the boundary between newly innervated and non-innervated areas of the womb (Piontelli, 2010). This (epistemic) exploration enables the fetus to become acquainted with and attuned to the statistical regularities of the environment to reliably deduce and optimize future actions to minimize expected surprise (i.e., uncertainty) and thereby maintain homeostasis (Friston et al., 2015). When we use touch to explore the external environment, it assists us in developing and establishing a baseline from which we can then develop our beliefs (and evaluate surprise or prediction errors in terms of deviations from baselines or prior beliefs). It is not surprising that touch has been suggested to be the first sense to develop *in utero* (Gottlieb, 1971).

Through exploration, the inherent need and desire for communication and differentiation between self and others can also be seen by prioritizing relevant sensory information for communication. All of these examples demonstrate the critical role of touch in comprehending and establishing a foundation for communication and a general understanding of oneself in relation to regulating homeostasis to their current external environment. By allowing for a shared experience with another and differentiating between self and others, self-awareness can be considered a precondition for developing synchrony. Even at 14 weeks gestation, twins can direct movements toward their co-twin in an attempt to communicate and co-regulate basic homeostatic needs in a self-organized manner through perception as action (Noë, 2004; Castiello et al., 2010).

### The Importance of Touch in Activating the Reward Pathways in Synchronous Interpersonal Relationships

Humans are distinct from many other species that reproduce, because human social interactions are not solely for practical development (feeding or protection) but to establish a social relationship to complete the self-fulfilling prophecy that everyone is alike—sometimes discussed in terms of social niche construction (Creanza and Feldman, 2014; Constant et al., 2019; Veissiere et al., 2019). Every 2–4 min, caregivers will instinctively copy and imitate up to 20% of the facial expressions of young infants, and infants will also physically copy their parents creating a degree of synchrony (Ray and Heyes, 2011; Slaughter, 2021). At six months of age, it is suggested that infants follow their partner’s gaze based on the promise, reward, and prediction that future stimulus of interest or social attention will occur in that direction, further suggesting the embedded desire to synchronize (Szufnarowska et al., 2014; Siposova et al., 2018). If the other party is interested and attuned, the individual is viewed as a valuable source of sensory information (that will resolve uncertainty), which helps the individual develop a sense of social understanding and connection to others; this is seen why infants are only satisfied when their partner matches their emotions (Liszkowski et al., 2004). Moreover, the new social connection and feeling understood reinforced by touch is seen as rewarding as it initiates the repetitive release of the endogenous peptides oxytocin and dopamine, as evidenced by research on skin to skin contact (Feldman, 2012; Seehausen et al., 2012).

The reward system is partly regulated by frontostriatal circuits, which respond to and connect reward, passion, and intention. The sophisticated higher-order cognitive processes needed to create human attachments comprises of frontostriatal connections in the reward system exerting top-down control over the qualities of trust, empathy, and commitment necessary to assign reward to developing and maintaining relationships (Pauli et al., 2016; Feldman, 2017). In romantic couples there is an increase in availability of u-opioid receptors in the reward centers because of touch (Nummenmaa et al., 2016). While ostensive cues such as touch are necessary for initiating communication, they would be deemed irrelevant in establishing a connection and alliance without the attention and intent embodied in the cue. In active inference, rewards are cast as prior preferences such that not securing a reward is surprising and therefore something to be avoided. On this view, uncertainty is inherently aversive in the sense it suggests a lack of precise beliefs about what to do and whether the outcome will be preferred. Indeed, empirical evidence suggests that dopamine reflects the degree of confidence in plans that lead to rewarding outcomes—or not (Friston et al., 2014; Schwartenbeck et al., 2015).

Babies are content with their communication partner only when their partner shares their intentions (Grewen et al., 2005). Otherwise, they become dissatisfied and cry when they do not receive an emotional response that matches theirs (Liszkowski et al., 2004; Carpenter and Call, 2013). This reflects AI in that the brain filters sensory information selectively to ensure that it corresponds to the individual’s priors, avoiding sensory input that does not match to try to avoid and correct prediction errors from unexpected cues (Pezzulo et al., 2015). Additionally, with repeated unsuccessful communications, the source could be deemed imprecise in collecting sensory evidence to support their specific existing prior, as well as predicting future encounters would be highly costly in regards to free energy causing the infant to be motivated to seek alternative sources minimizing unexpected cues (Fonagy and Allison, 2014; Friston and Frith, 2015a; Vasil et al., 2020).

With proximity to caregivers, the infant is motivated to regulate allostatic control, promoting growth and physiological, neuropsychological, and behavioral development (McGlone et al., 2017). The sudden dissatisfaction and decision to ignore them can be interpreted as a rapid error correction mechanism designed to halt unexpected stimulus and avoid repeating this failed interaction and ensuing uncertainty. By minimizing prediction errors and biophysical surprises, the individual creates a self-fulfilling prophecy consisting of action-perception cycles which maximizes the evidence for their own personal inner or generative model of the world they live in Vasil et al. (2020)—something that is sometimes referred to as self evidencing (Hohwy, 2016). This increased brain–brain synchrony and decreased (expected) surprise will stimulate the reward pathways in the limbic system, responsible for the design, and regulation of critical survival and motivational processes, thereby promoting a more enjoyable (i.e., predictable) social life (Sokolowski and Corbin, 2012).

### The Role of Touch in Overcoming Uncertainty, Increase Saliency and Inferring Hidden Thought

To synchronize and overcome uncertainty, we frequently rely on and employ specific perceptual and ostensive cues such as gaze, touch, and facial expressions to elicit an individual’s attention to communicate with them and establish new interpersonal connections (Vasil et al., 2020). A gentle caregiving stroking motion activates the CT fibers which is believed to contribute to the modulation of an individual’s silhouette and more specifically the face, thus helping self-other differentiation, whilst also increasing subjective self recognition and motivation for cooperative communication (Panagiotopoulou et al., 2017; Della Longa et al., 2019). This is believed to be responsible for 4-month-old infants recognizing faces even when they are not looking directly at the face. This capability was previously absent compared to other forms of touch (Della Longa et al., 2019). Additionally, young children can infer that their partner attempts to communicate with them when they receive ostensive looks. This observation implies that gentle touch facilitates the initiation, mediation, and consolidation of social processing and alleviates anxiety (Brauer et al., 2016). Another example is mothers touching their infants for 65% of their first year of life, effectively bombarding them with sensory evidence. This will aid in regulating allostasis via interoceptive AI by invoking specific hierarchical precision weighted predictions and prediction errors to achieve homeostasis (Stack and Muir, 1990; Mateus et al., 2021). Observing touch or experiencing precise tactile behaviors assists individuals in accurately identifying another’s emotional state including anger, love, sympathy, fear and gratitude with up to 83% accuracy, thus increasing the benefit of touch including analgesia (Hertenstein, 2002; Hertenstein et al., 2006b; Goldstein et al., 2016). Moreover, reduced pain has been recorded when one feels they have been understood by another, a phenomenon arguably modulated by activity within the reward system and the cingulate cortex (Eisenberger, 2012; Oishi et al., 2013; Jensen et al., 2014). At three months, infants begin to respond to their mothers’ affectionate touch, gradually developing the ability to conduct intentional affectionate touch themselves (Feldman and Eidelman, 2004).

Additionally, touch improves accuracy in inferring other hidden states, lowering the prediction error required to maintain homeostasis and predict behaviors, frequently resulting in synchrony and increased (joint) attention. Constant stimulation effectively bombards them with sensory evidence, assisting in regulating allostasis and fostering a strong bond. Infants rely on caregivers to assist them in achieving homeostasis through interoceptive AI, which involves evoking and creating specific hierarchical precision weighted predictions and prediction errors (Stack and Muir, 1990; Mateus et al., 2021). In this setting, precision scores the predictability of predictions and prediction errors, with increased precision weighting of belief updating under a shared narrative encourages (mutual) epistemic trust that may contribute to the development of the TA (Fonagy and Allison, 2014).

When we experience something for the first time it is often natural for us to explore the environment physically, as evolutionarily touch is the first sense to develop and therefore the primary contact point to the external world (Ciaunica, 2017; Ciaunica and Crucianelli, 2019). Moreover, it has been suggested that touch is considered fundamental in mediating and developing perceptual experiences in both interceptive and exteroceptive natures, to our self-embodiment and ownership within the external world we live in, in order for us to develop priors of the world around us (Gallace and Spence, 2010; Ratcliffe, 2013; Ciaunica and Crucianelli, 2019). We frequently initiate, mediate, and solidify social processing through physical touch, thereby alleviating social anxiety. This is especially true when confronted with more complex or unfamiliar policies; we instinctively seek additional sensory information to alleviate uncertainty, frequently touching objects to confirm their authenticity. Additionally, by acquiring additional information, we increase the likelihood of the preferred stimuli occurring and our sensitivity to it. This is demonstrated when newborns instinctively reach out to seek their mother’s breast (breast crawling), thereby increasing opportunities for social communication and the development of social priors while reducing uncertainty and minimizing expected surprise. Subsequently, the infant will benefit from the critical action of acquiring necessary nutrients and maintaining homeostasis (Uvnäs-Moberg et al., 2015).

#### The Role of Touch in Overcoming Uncertainty Through Creating Safety

A secure attachment to an individual is thought to develop due to prolonged skin-to-skin contact between mother and child; one good example is breastfeeding. Additionally, the fundamental need to feed to manage an infant’s homeostatic needs emphasizes the relationship’s importance. One could argue that the sense of security stems from the knowledge that the infants’ homeostatic requirements will be met (Bystrova et al., 2007). Touch is frequently used to regulate and alleviate stress by activating the neural networks associated with stress and lowering cortisol levels and increasing hippocampus cell development (Miles et al., 2006; Coan et al., 2013). When combined with periods of touch, a positive relationship frequently reduces both the heart rate and level of anxiety of the individual.

#### The Role of Touch in Empathy

While there is debate over the exact definition of empathy, it has been described as understanding others states by activating the individuals own personal, neural, or mental representations of the same state and communicating it to them (Fan et al., 2011; De Waal and Preston, 2017; Schaefer et al., 2021). A recent theory explaining the shared representations of perception and the subsequent motor action behavior proposes a perception-action model of empathy, which builds on the suggested involvement of a person and goal-specific bottom-up overlap between motor and affective representations of self and other. Arguably, this may contribute to understanding others through empathy and embodiment (Preston, 2007; De Waal and Preston, 2017; Rizzolatti and Caruana, 2017; Schaefer et al., 2021).

Moreover, it can also be expanded to explain the distributed representations developed through the observers’ previous memories, priors and connotations for the target and situation creating variations to the degree individuals empathize with others (Preston, 2007; Preston and Hofelich, 2012; De Waal and Preston, 2017). Additionally, touch and tactile acuity have been recently found to directly contribute to the feelings of sympathy for others and activate the somatosensory cortices most likely via the insula, thus suggesting a contribution to accurately defining our own emotions and mental states aswell as others (Schaefer et al., 2021). Moreover, empathy proposes that the person care about the other individual and wants to help which is often reinforced and exemplified with physical touch. Individuals with empathic tendencies have been suggested to contribute to good therapeutic alliances and beneficial treatment outcomes (Goldstein et al., 2016, 2018).

Empathy can be considered the motivation behind the prosocial response of comforting touch (Romero et al., 2010). Empathy as an emotion is a way of relating to the environment and sharing feelings and higher-order concerns with another to regulate them (Frijda and Mesquita, 1994). Empathy has even been shown to predict the amount of analgesia one can experience from touch when the sensory aspects of the pain matrix are activated (Goldstein et al., 2016). Indeed, the activation of similar neural networks during observation, particularly in the pain-neomatrix and related motivational regions of the anterior mid-cingulate cortex and anterior insula, when directly experiencing the situation to observing a similar situation suggests we are able to understand and share others’ emotions by partially processing them within our own emotional systems (Jackson et al., 2005; Vogt, 2005; Kilner et al., 2007; Friston et al., 2011; Lamm et al., 2015).

The same neural activation in the inferior parietal lobe and IFG was found in both the target and empathizer on hand-holding during the perception of noxious stimulus suggesting that the IPL facilitates the shared experience to both parties (De Waal and Preston, 2017; Korisky et al., 2020). Moreover, the involvement of the IPL has also been suggested as play an important role in the mechanism to infer another’s intentions and thoughts based on one’s observations, which may allow us to reevaluate the adverse stimulus to create an analgesic effect (Kilner et al., 2007; von Mohr et al., 2018). Furthermore, the more empathetic an individual is to another the higher the degree of brain-to-brain synchrony (Reddan et al., 2020). This is reflected in the higher the clinicians’ precision at inferring a patient’s pain level, the more positive analgesic effect the patient receives from the treatment possible due to the synchrony involved in adaptive empathic touch (Ellingsen et al., 2020; Shamay-Tsoory and Eisenberger, 2021).

Through social coordination, the increased physical multimodal sensory nature of touch on a common threat result in increased bonding and activation of the ACC. Increased ACC activation has strong links to the amygdala and increases motivation and reward pathways in the ventral striatum reinforcing why gentle touch is commonly selected to express empathy (Barrett and Fleming, 2011). Thus, empathy in conjunction with touch can aid in forming synchrony and coupling, as demonstrated by the alpha-mu band, which is also involved in pain perception and empathy, particularly in the frontocentral regions (Morelli et al., 2014). Moreover, comforting touch including hand holding can decrease the activity in the pain regions such as the dorsal ACC and anterior insula resulting in an analgesic effect (López-Solà et al., 2019; Shamay-Tsoory and Eisenberger, 2021). Hand holding also increases the association between the pain network, somatosensory cortices, and the default mode network (López-Solà et al., 2019). Empathy has also been linked to an individual’s degree of parent-infant synchrony earlier in life (Feldman and Friston, 2010).

#### Repetition as a Tool to Overcome Uncertainty

Physical repetition can further enhance saliency, precision, and synchrony. The Nso culture, based in rural Bamenda grass fields in Cameroon, primarily communicates with their infants through proximal parenting, emphasizing empathic body contact and synchronized rhythmic rocking (Kartner et al., 2010). This physical parenting method promotes self-awareness, compliance, and the development of priors, resulting in a more symbiotic relationship (Keller et al., 2004, 2005). As the bond becomes more familiar and predictable, the corticostriatal connectivity within the striatum shifts from ventral to the dorsal striatum (Tops and Boksem, 2012). Increased awareness of a preceding or stimulating event is frequently regarded as necessary for synchrony and shared experience. Increased attention to the stimulus facilitates future activation of the prior.

Manzotti et al. (2019) demonstrated that dynamic stroking bilaterally activates the CT fibers and posterior insular cortex in mothers and infants, assisting individuals in developing the ability to regulate, synchronize, and anticipate/prefer specific sensory stimuli. The repetition aids in enhancing saliency, precision, synchrony, and coupling in the appropriate temporal lobe. Individuals develop an expectation and preference for specific communicative cues or movements due to repeated exposure, resulting in embedded automatic priors, learned prediction errors, and future policies (Romberg and Saffran, 2010; Friston and Frith, 2015b). Additionally, the coupled action-perception cycle increases more precise policy mappings, frequently originating from social behaviors and initiating a downward modulation via automatic policies. By developing these automatic policies via behavioral and neural synchrony, the brain can become more efficient at maintaining homeostasis (i.e., avoiding surprises) while avoiding (computationally and metabolically) costly changes to beliefs. The brain assumes it has chosen the correct policy to regulate allostasis (Constant et al., 2018; Koban et al., 2019). Confirming their priors more rapidly results in a more prominent response to achieving homeostasis, as evidenced by changes in heart rate and anxiety (Smith et al., 2014). Touch can be used as an ostensive cue through repeated imitation of facial expressions by a mother, which results in the infant making the expression over 500% more often. As a result, it is reasonable to expect a similar response in other situations that involve repeated affection touch.

### Adaption of Priors to Achieve Synchrony

Individuals develop similar—but not identical—policies due to learning and continually updating their actions via communicative coupling with the mental states of others. This phenomenon is demonstrated by Bannard et al. (2009), who found that when two 3-year-old infants communicate, the utterances and grammar imputed between the two individuals are reflected at a rate of 59 and 63%, respectively. Everyone’s neuronal dynamics are chaotically coupled by inferring the others’ hidden mental states, resulting in a bidirectional flow of sensory information synchronizing with their prior beliefs. This process gradually increases the expectation, attention, and evidence accumulation that underwrite belief updating in the brain—and the implicit revision of prior beliefs and conceptual or generative world models (Vasil et al., 2020). The canonical loop of coupled action-perception cycles is the process by which dyads generate and modify cycles until they achieve the end goal of aligning their mental states and communicative exchanges (Friston and Frith, 2015a). It is a deliberate process of creating action and perception loops that reliably connect the two members of the dyad until they become entwined via a shared interpretation of their exchanges; i.e., a shared narrative or simply, “singing from the same hymn sheet.” This can be explained by AI as a simple consequence of trying to make the world more predictable; namely, less surprising (Friston et al., 2015).

### Adaptation to Experts Based on Hierarchical Structure

Infants frequently communicate hierarchically, relying on and basing social interactions on the sensory information provided by older individuals deemed reliable sources of sensory information with fewer, more precise prediction errors and more experienced priors. Due to their inexperience and lack of prior knowledge, infants rely heavily on sensory information such as touch to form and solidify their developing beliefs through an “interrogative motivation” based on the premise that everyone is the same (Karmali et al., 2018). Infants constantly create situations where they can request additional information, for example, by pointing to resolve their uncertainty and gather additional information to solidify potential beliefs, thereby activating their subcortical reward centers (Lucca and Wilbourn, 2018).

Asymmetric entrainment is the process of adapting existing priors to someone else’s more precise and reliable priors around a particular niche to synchronize (Brownell, 2011). They then reduce their concerns, free energy, and uncertainty while increasing sensory information to confirm or adapt their pre-existing priors to more likely precise priors, frequently those of their caregivers and mentors (Friston and Frith, 2015b). By adapting their current prior beliefs to align with those of an experienced adult, they can raise the precision weighting, reliance, expectation, and information flow associated with their prediction errors to the same level as their partner, resulting in symmetrical coupling and synchronization (Moran et al., 2013; Hasson and Frith, 2016). Adults’ increased precision-weighting will result in symmetric coupling, causing a shift in their priors and low confidence in their top-down beliefs, as well as aiding in the dismissal of alternative prior explanations, confirming their self-fulfilling prophecy (Powers et al., 2016). As a result of this new sensory input, their previous prior policies will be enhanced and selected with greater precision or confidence in the future (Kemp et al., 2007).

With age, the infant develops a more precise inner interoceptive model of the world, which is enhanced when combined with touch. Physical touch is critical for an infant’s self-regulation development or homeostasis possibly due to increased ability for emotional regulation through the increased activation in the prefrontal cortex (Shamay-Tsoory and Eisenberger, 2021). As a result of the interoceptive prediction errors generated by the pre-frontal cortex, priors will be updated in a multimodal homeostatic manner, satisfying multiple skeletal motor reflex arcs (Smith et al., 2014).

This is demonstrated by cardiac function and heart rate synchrony, which activate the somatosensory cortex and insula, reflecting interoceptive inference about bodily states. The somatosensory system is involved in cognitive maturation and is within the mirror neuron system, which is required for individual synchrony and social coordination. This underwrites interoceptive and affective inference under a hierarchical generative model that has to generate predictions of bodily sensations (Seth, 2013; Gu and FitzGerald, 2014; Smith et al., 2014; Pezzulo et al., 2015; Fotopoulou and Tsakiris, 2017; Allen et al., 2019). Heart rate variability is an example of allostatic regulation. When an infant reaches 9 months of age and can comprehend intersubjectivity, a shift occurs from infant-led mother follow synchrony to an increasingly brief mutual moment to moment approach with both responding to the other (Feldman, 2007). While they may temporarily increase their HRV to reduce predictive error, they will quickly revert to their developed baseline reference (Suga et al., 2019). The fluctuations in physiological synchrony from moment to moment are unsurprising, as it is an active process primarily activated via touch. The levels fluctuate in response to external stimuli such as priors, mood, and stimulation, with research indicating that social understanding occurs via communication intent. Multiple internal mechanisms facilitate this process, with the brain discarding sensory input that does not correlate with its internal model to forecast the current state. Additionally, it reflects the homeostasis and changes in the reliability of sensory information and the influence on prediction errors, attend weighting associated with the context and existing priors (Feldman and Friston, 2010). This is seen in the brain-to-brain synchrony between teachers and students, which was higher when increased joint attention through face-to-face interactions, gaze, ostensive cues, physical gestures or activities, as they become more in tune with their surroundings compared to a traditional lecture environment.

## Mechanisms Increasing Saliency Within Synchrony

**FIGURE 1:**
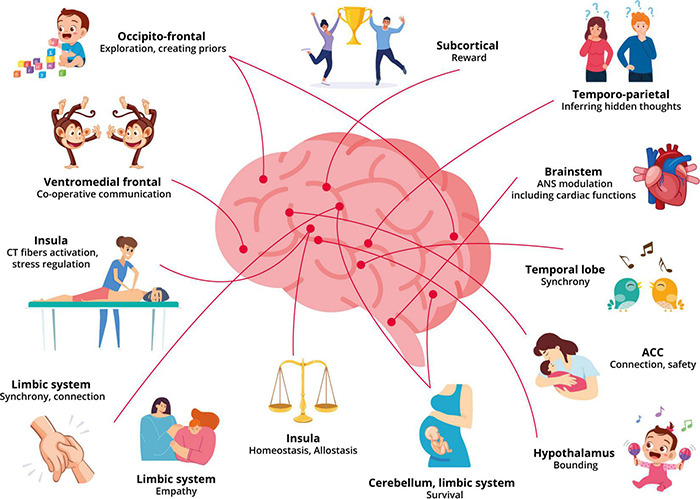
An overview of the effects of touch and therapeutic alliance on the different networks of the brain.

Repetitive stimulation of the CT afferents in touch increases the number of oxytocin receptors and results in the release of endogenous peptides with tighter crosslinks such as dopamine, oxytocin, and opioids; all while regulating and synchronizing the autonomic system (Löken et al., 2009; Ackerley et al., 2014; Pawling et al., 2017). The more compact crosslinks aid in the memory of the relationship and context-specific patterns that stimulate a prior. Moreover, multimodal sensory stimulation including tactile may aid in the increase of cortical oxytocin levels through its involvement in the experience-dependent development of social cortices in addressing conditions with reduced social interaction, potentially increasing trust, empathy and face memory (Green and Hollander, 2010; Yamasue et al., 2012; Zheng et al., 2014). The oxytocinergic projections from the paraventricular nucleus regulate regions of the brain involved in social responsiveness, social recognition, increased bonding, pain and stress reduction (Kumsta and Heinrichs, 2013; Uvnäs-Moberg et al., 2015; Carozza and Leong, 2021). Moreover, increased oxytocin levels are associated with a reduction in neural activation in the anterior insular and increased activation in the prefrontal cortex further contributing to the reduction in unpleasantness (Kreuder et al., 2019). Additionally, the oxytocin pulses released during skin-to-skin contact or breastfeeding are longer lasting than those released during labor, which is a more intense but brief extreme event. As a result, it demonstrates that the brain prioritized the previously desired connection and regulation of allostasis following an intense emotional connection. It is also reflected in the frequent and cherished long-term relationships that develop between close friends, lovers, and family members due to shared experiences, habits, and trust (see Figure 1 for an illustration of the neurobiology underpinning our model). Moreover, the role of a practitioner to help regulate the patient’s allostasis, increasing connection and trust that have been suggested to be significant components of increasing and developing brain-to-brain synchrony, also overlap with the components of a beneficial TA.

The restructuring of neural pathways involved in choice and behavior is affected by the interaction of oxytocin and dopamine in the striatum, which provides the substructure for bonding, especially when motivation and intention are present (Kim et al., 2014; Feldman, 2017). This can be translated over time to provide a habituated drive toward elevated oxytocinergic levels, affecting how infants communicate with others. This is demonstrated in depressed mothers, who produce less oxytocin, milk, and face-to-face interactions with their children. Additionally, there is a greater risk of the child developing social insecurities later in life. This is demonstrated by the termination of the alpha response in the posterior superior temporal sulcus, which results in the cessation of top-down processing necessary for empathic resonance, and by decreased glucocorticoid receptor density and neurogenesis in the hippocampus, which correlates with decreased empathy (Jones et al., 1997; Heim et al., 2004; Stuebe et al., 2013; Pratt et al., 2017). However, through massage type stroking an increased glucocorticoid receptors and reduced methylation in the hippocampus that regulates insulin like growth hormone is found (Baldini et al., 2013; Murgatroyd et al., 2015).

We can speculate through AI that the context in which an infant develops their first priors will influence their subsequent actions and high-level actions later in life as they acquire the deontic value of policies (Ainsworth et al., 1978; Ramstead et al., 2016). This is in addition to the pulsating nature of oxytocin release during attachment, which appears to be established during infancy. All subsequent attachments will be organized into dyad-specific temporal rhythms based on their prior attachment experiences during their youth to permanently embed them (Feldman, 2017; Shimon-Raz et al., 2021). This can be seen in prairie voles, where the density of oxytocin receptors predicts the amount of time spent huddling with their partner and grieving over their mate later in life (Bosch et al., 2016). Additionally, it suggests that elevated oxytocinergic levels in childhood will influence communication mechanisms and preferences later in life, reiterating the critical role of affectionate touch, particularly on infants, in developing cooperative communication and synchrony.

## Conclusion

**FIGURE 2:**
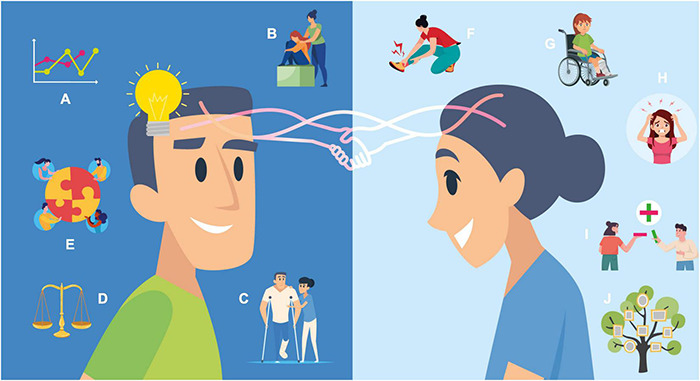
Therapeutic alliance as active inference. **(A)** Opportunity to build a synchronous relationship and understanding of the injury. **(B)** Opportunity to show empathy to reduce anxiety and fears. **(C)** Working together to create a therapeutic alliance for recovery. **(D)** Regulating disrupted allostasis. **(E)** Joint attention to regulate allostasis and support recovery. **(F)** Injury and pain—“what have I done?” **(G)** Fears of what the injury will become. **(H)** Anxiety over the injury. **(I)** The hope of collaborative communication to understand and create a treatment and management plan. **(J)** Previous priors surrounding the injury including injury beliefs, social expectations, family and injury history.

We have proposed a framework in this article to explain the critical role of touch in developing therapeutic alliances and synchrony in general (Figure 2). Touch appears to be critical in initiating, developing, and enhancing the salience of synchronous relationships. This framework is based on active inference and encompasses an integrative hierarchical framework that promotes synchrony and the therapeutic alliance to engender allostasis. While it has long been recognized that touch and skin contact have therapeutic benefits for patients and individuals, much less research has been conducted on the bidirectional role of touch. This generic model explains how touch can be used to infer and predict others’ states of mind, which are crucial for developing dyadic and triadic relationships in general and in a clinical setting. However, it is important to note that there is a particular emphasis on perinatal and pediatric care, due to its focus on the role of touch and developing synchrony. Additionally, by inferring individuals more precisely, we can help create more adaptive feedback loops that minimize surprise, increase understanding, and reduce physical and psychological stress, all of which are crucial for daily survival. The ensuing model considers how touch can strengthen and tighten neurobiological and social bonds to achieve a synchronous relationship and a more effective therapeutic alliance.

Future research will contribute to solidifying the potentially broad bidirectional neurobiological coupling implied by the role of touch between two individuals. Additionally, it may assist in validating each of the predictive steps involved in the dynamic interaction between practitioner and patient, thereby advancing the therapeutic alliance critical to treatment success. Additionally, establishing the role of touch paves the way for explaining the complex and dynamic interactions that occur in touch-based therapies. Additionally, it will aid in the development of an understanding of the barriers encountered during interactions with patients who are cognitively incapable of updating their priors and the factors that may or may not be influenced by comforting touch.

## Author Contributions

ZM wrote the first draft of the manuscript. All authors contributed to the conception and revision of the manuscript and approved and are accountable for the submitted manuscript.

## Conflict of Interest

JE was employed by Malta ICOM Educational Ltd, Malta. The remaining authors declare that the research was conducted in the absence of any commercial or financial relationships that could be construed as a potential conflict of interest.

## Publisher’s Note

All claims expressed in this article are solely those of the authors and do not necessarily represent those of their affiliated organizations, or those of the publisher, the editors and the reviewers. Any product that may be evaluated in this article, or claim that may be made by its manufacturer, is not guaranteed or endorsed by the publisher.
